# The gut microbiome: an emerging epicenter of antimicrobial resistance?

**DOI:** 10.3389/fmicb.2025.1593065

**Published:** 2025-05-20

**Authors:** Soham Pravin Deshpande, Swathi Sujith, Renitta Jobby, Satish Kumar Rajasekharan, Vinothkannan Ravichandran, Adline Princy Solomon

**Affiliations:** ^1^Amity Institute of Biotechnology, Amity University Maharashtra, Mumbai, India; ^2^Quorum Sensing Laboratory, Centre for Research in Infectious Diseases (CRID), School of Chemical and Biotechnology, SASTRA Deemed to be University, Thanjavur, India; ^3^Amity Centre of Excellence in Astrobiology, Amity University Maharashtra, Mumbai, India; ^4^Department of Biotechnology, School of Bioengineering, SRM Institute of Science and Technology, Chennai, India; ^5^Amity Centre of Excellence in Drug Discovery and Development, Amity University Maharashtra, Mumbai, India

**Keywords:** gut microbiome, antimicrobial resistance, multidrug resistance, screening of AMR, microbiome

## Abstract

The human gut is one of the most densely populated microbial environments, home to trillions of microorganisms that live in harmony with the body. These microbes help with digestion and play key roles in maintaining a balanced immune system and protecting us from harmful pathogens. However, the crowded nature of this ecosystem makes it easier for harmful bacteria to acquire antimicrobial resistance (AMR) genes, which can lead to multidrug-resistant (MDR) infections. The rise of MDR infections makes treatments harder, leading to more extended hospital stays, relapses, and worse outcomes for patients, ultimately increasing healthcare costs and environmental strain. Since many MDR infections are challenging to treat, nosocomial infection control protocols and infection prevention programmes are frequently the only measures in our hands to stop the spread of these bacteria. New approaches are therefore urgently required to prevent the colonization of MDR infections. This review aims to explore the current understanding of antimicrobial resistance pathways, focusing on how the gut microbiota contributes to AMR. We have also emphasized the potential strategies to prevent the spread and colonization of MDR infections.

## 1 Introduction

The gut microbiome, a complex ecosystem in the gastrointestinal tract, harbours trillions of commensals, symbiotic organisms, including bacteria, viruses, fungi, archaea, and eukaryotes. These organisms contribute intestinal integrity, immunity, metabolism, digestion, mental health, and pathogen defence to the host (Anto and Blesso, 2022; Lane and Yadav, 2020). The microbial composition of each individual is unique and stable, but the significant phyla remain the same, and an individual will conserve over 60% of the gut microbial phylotypes for 2 years (Manichanh et al., 2010). The microbiome encodes nearly three million genes that produce hundreds of metabolites, outnumbering the roughly 23,000 genes in the host genome (Valdes et al., 2018). However, this ecosystem can serve as a reservoir and epicenter for developing antimicrobial resistance (AMR).

AMR, a global health concern of since the mid-20th century, is the development of resistance by microorganisms to the antimicrobial medications that are used to treat them, reducing clinical efficacy and increasing treatment costs (Penders et al., 2013; Prestinaci et al., 2015). Since antibiotics are not pathogen-specific and are prescribed to treat infections leading to overdose, they impact commensal microbiota present in the same habitat (Bag et al., 2019; Llor and Bjerrum, 2014). The continuous emergence of resistant genes and mechanisms contributes to the global spread of AMR. Several diseases previously treated successfully, with any of the several drug classes have developed resistance, making it difficult to inhibit their growth. The emergence of resistant pathogens such as methicillin-resistant *Staphylococcus aureus* (MRSA), penicillin-resistant and macrolide-resistant *Streptococcus pneumoniae*, carbapenem-resistant *Enterobacteriaceae*, third-generation cephalosporin-resistant *Klebsiella pneumoniae*, cephalosporin-resistant *Escherichia coli*, carbapenem-resistant and multidrug-resistant *Pseudomonas aeruginosa*, have all been classified by the World Health Organization as high or critical priorities for the development of new antibiotics (Kessler et al., 2022). AMR is developed by selecting resistant characteristics, which permits organisms to survive and reproduce, resulting in the persistence of resistant populations (Ferri et al., 2017). Resistance arises because of genetic mutation and horizontal gene transfer. Horizontal gene transfer, a prevalent mechanism, occurs due to the acquisition of resistance genes from environmental and microbial reservoirs. Bacteria employ various mechanisms to achieve antimicrobial resistance namely use of efflux pump, altering the antibiotic target (for example, by altering binding sites in ribosomal RNA), reprogramming metabolic pathways, and production of enzyme to inactivate the antibiotic (Wright, 2005). Antibiotic resistance affects both developed and developing nations equally, therefore, it is essential to examine how antibiotic resistance is spreading over the world. The widespread use of antibiotics in hospitals, the general population, and agriculture has increased the stress on selection, leading to the persistence of resistant microbes in high-income nations, necessitating shifting to more expensive, broad-spectrum antibiotics. The need for antibiotics is rising in low- and middle-income nations due to increased incomes, a more significant hospitalisation rate, and a high prevalence of hospital infections (Laxminarayan et al., 2013). By 2050, according to estimates, antibiotic resistance will cause almost 10 million deaths annually and a loss of $100.2 trillion in GDP (Chokshi et al., 2019). It is essential to investigate the key socioeconomic and political factors that influence how quickly AMR spreads in both developed and developing nations (Chokshi et al., 2019). The direct monetary effects of AMR on health care are high expenses associated with expensive and intensive treatments and an increase in resource consumption (Dadgostar, 2019).

Numerous *in silico* metagenomics studies have confirmed that the human gastrointestinal tract acts as a reservoir for AMR genes, capable of transferring these genes to transient, pathogenic bacteria (Bag et al., 2019; Cheng et al., 2012; Radovanovic et al., 2023; Ghosh et al., 2013). The spread of antibiotic resistance genes (ARGs) is increased in international human interaction, wherein antibiotic-resistant bacteria from one part of the world are swiftly transferred and spread to far-off nations at great geographic distances (Okeke and Edelman, 2001). Since the development of antibiotic resistance increases the probability of therapeutic failure, relapses, extended hospital stays, and poorer clinical outcomes, treating infections caused by multidrug-resistant organisms provides a significant clinical challenge (Gargiullo et al., 2019). Determining the antimicrobial resistome of the human gastrointestinal microflora will, therefore, be of great importance in evaluating the process of resistance genes being transferred among intestinal microorganisms. The ways through which mutualistic and pathogenic bacteria in the human gut potentially exchange antimicrobial resistance genes have been investigated in the current review.

## 2 Human gut microbiome

The gut microbiome, plays a vital role in the overall wellbeing of the individual, consists of, principally, of five significant phyla of distinct and complex colony of microorganisms. Firmicutes include *Lactobacillus*, *Bacillus*, *Clostridium*, *Enterococcus*, and *Ruminococcus* (Kho and Lal, 2018; Rinninella et al., 2019). *Bacteroidetes* include *Bacteroidia*, *Flavobacteria*, *Sphingobacteria*, and *Cytophagia* (Thomas et al., 2011). *Actinobacteria* include *Corynebacterium*, *Propionibacterium*, *Rothia*, *Actinomyces*, and *Bifidobacterium* (Wu, 2013). *Proteobacteria* include *Escherichia coli*, *Salmonella*, and *Campylobacter* (Moon et al., 2018). *Verrucomicrobia* is primarily represented by *Akkermansia muciniphila* (Dubourg et al., 2013).

The human gastrointestinal tract (GI tract), with a surface area of 250–400 m^2^, forms an interface between the host, environmental factors, and antigens. Over the course of a lifetime, the human GI tract processes about 60 tonnes of food and encounters various pathogens that can be detrimental to gut health (Thursby and Juge, 2017). Initially, the newborn gut is aerobic, but the first colonizers, facultative anaerobes, create a new environment with a low level of oxygen, beneficial for the growth of anaerobes such as *Bacteroides*, *Clostridium*, and *Bifidobacterium* spp. (Breitbart et al., 2008; Fouhy et al., 2012; Rodríguez et al., 2015). The sources of this diversity of gut microbes include nutritional, environmental, and maternal factors, gestational age, delivery technique (vaginal birth vs. assisted delivery), feeding (breast milk vs. formula), sanitation, and antibiotic use (Rodríguez et al., 2015; Townsend et al., 2021). Studies show that vaginal delivery exposes newborns to maternal vaginal microbiota (primarily *Lactobacilli*), whereas caesarean sections result in significantly different microbial populations (McCann et al., 2018; Firoozeh and Zibaei, 2019). By human anatomy, exposure to the mother’s faecal microbiota after birth is a significant means of transmission. One of the bacterial species with the highest likelihood of direct transmission from mother to newborn through faeces is *Enterobacteriaceae* (Rodríguez et al., 2015). Mother’s breastmilk acts as a vehicle for the vertical transmission of *Bifidobacterium*, *Streptococcus*, and *Staphylococcus* (Hunt et al., 2011).

The structure, diversity, and functional capabilities of the newborn microbiota increase and resemble those of the adult microbiota by the time the child is 2.5 years old, with temporal patterns that are unique to each newborn (Eckburg et al., 2005; Bäckhed, 2011; Firoozeh and Zibaei, 2019). The *Bacteroidetes* phylum and *Clostridium* cluster IV are more prevalent in those over 65 than in younger people (Claesson et al., 2011).

Furthermore, the composition of gut microbiome varies significantly among individuals due to genetics and environmental factors such as routine habits, dietary pattern, personal hygiene, health, medications such as antibiotics, and the use of prebiotics and probiotics (Ahmad et al., 2019; Cunningham et al., 2021). Diet is considered one of the key factors affecting the composition of an individual’s microbiota irrespective of age. Seasonal variation in the gut microbiome, influenced by the consumption of fresh foods, leads to shifts in composition, with *Bacteroides* common in summer and *Actinobacteria* in winter, indicating the influence of complex carbohydrate intake on microbiome plasticity (Davenport et al., 2014). Furthermore the composition and heterogeneity can be altered in case of obese and nonobese individuals, where more *Firmicutes* and fewer *Bacteroidetes* were observed in obese compared to non-obese adults (Pinart et al., 2021). The composition varies widely among ethnic groups and provides more information about the individual influenced by the same geographical area (Deschasaux et al., 2018; Gaulke and Sharpton, 2018; Schnorr et al., 2014). The gut microbiome greatly influences the health, brain, well-being, stress, and anxiety. Social interactions increase the diversity of the microbiome while anxiety and stress decrease the same (Johnson, 2020).

The gut microbiota coevolved with humans and maintains host health by regulating metabolism, physiology, and immune functions (den Besten et al., 2013; Natividad and Verdu, 2013; Singh et al., 2019). According to estimates, the human microbiota contains roughly 10^14^ microbial cells, with a microbial cell-to-human cell ratio of 1:1 (Sender et al., 2016; Thursby and Juge, 2017). Colonic bacteria produce carbohydrate-active enzymes that convert complex carbohydrates into short-chain fatty acids (SCFAs) like propionate, butyrate, and acetate (Louis et al., 2014; Thursby and Juge, 2017). These SCFAs are absorbed by epithelial cells, regulating gene expression, inflammation, and cell proliferation (Corrêa-Oliveira et al., 2016). Gut anaerobes create acetate, whereas *Bacteroidetes* and *Firmicutes* synthesis propionate, butyrate through glycolytic and acetyl-CoA pathways, as well as succinate or propanediol pathways (Louis and Flint, 2009; Louis and Flint, 2017; Macfarlane and Macfarlane, 2003; Morrison and Preston, 2016). Variations in the composition of the gut microbiome can endanger human health, indicating its critical role in human health (Vandenplas et al., 2020) (see Table 1).

**Table 1:** Factors influencing gut microbiome.

S. No.	Factor	Description	Impact on AMR and host	Examples	References
1.	Diet	Nutrient intake	Change in microbial community protect from inflammations and non-infectious colonic diseases	Fiber diet, plant or animal based	David et al. (2014), De Filippo et al. (2010), and Wu et al. (2011)
2.	Age	Microbiome changes across lifespan	Different microbiota depending upon age	Infant vs. young vs. elderly	Ghosh et al. (2022) and Li et al. (2024)
3.	Health status	Presence of diseased condition	Maintaining homeostasis, promotes overall health	Inflammatory bowel disease and metabolic disorders	Afzaal et al. (2022) and Shreiner et al. (2015)
4.	Geographical location	Regional differences	Change in microbial diversity	Urban vs. rural areas, western	Gaulke and Sharpton (2018)
5.	Sanitation and hygiene	Access to clean water and sanitation facilities	Shift in microbial diversity	Hand wash	Monira et al. (2023)
6.	Exposure to antibiotics	Misuse of antibiotics	Selects for resistant strains, reduces diversity	Use of tetracycline, amoxycillin, influencing overall microbial community resilience	Nhu and Young (2023)
7.	Lifestyle factors	Habits and behaviors affecting microbiome	Modify the microbial diversity	Smoking, alcohol consumption, sleep deprivation	Ren et al. (2023)
8.	Genetic factors	Genetic makeup of host influence microbiome composition and function	Genetic predisposition to harbour certain resistant strains	Variations in immune response genes	Blekhman et al. (2015)
9.	Immune system	Immune response of the host	Influence the microbial composition	Inflammatory responses, immune tolerance	Zheng et al. (2020)
10.	Mode of delivery	Vaginal or caesarean	Influences the initial gut colonization	*Bifidobacterium*, *Enterococcus* spp.	Brinkac et al. (2017), Wen and Duffy (2017), and Zhang et al. (2021)
11.	Feeding method	Breastfeeding or formula	They are one among the first microbes to enter the infant’s body, and they could play an important role in health	Breast milk contains potential probiotic bacteria and IgA antibody	Davis et al. (2022) and Wen and Duffy (2017)
12.	Gender	Biological differences	Differences in microbial diversity and composition	Hormones influence the microbiota	Niemela et al. (2024) and Yoon and Kim (2021)

## 3 The gut microbiome and antimicrobial resistance

Antibiotic resistance, a severe threat to public health, signals the end of an era of antibiotics as a “golden therapy” and returns us to a time when effective treatments for microbial infections existed (Huddleston, 2014). Infectious disease remains one of the primary causes of death worldwide, pharmaceutical companies have slowed the drug development process, providing only 0.2% of new drugs (Spellberg et al., 2004). Bacteria develop resistance through mechanisms such as horizontal gene transfer, overexpression of efflux pumps, and protection of the drug target site by designing a specific protein (Munita and Arias, 2016).

The gut microbiome, essential for host wellbeing and a reservoir for ARGs are disrupted by dietary modifications, stress, antibiotic use, causing microbial dysbiosis, having detrimental effect on health and reduces resistance to pathogen colonization (Singh et al., 2019; Gargiullo et al., 2019).

The human gut microbiome, which houses 3.3 million non-reductant genes, is estimated to be 150 times larger than the human host (Qin et al., 2010). The confined environments of the diverse microbiome provide favourable conditions for genetic exchange between transitory and resident bacteria, as well as resident microbes (Brinkac et al., 2017). AMR genes in the gut, collectively termed the resistome, are categorized as intrinsic and mobile (Gargiullo et al., 2019; Singh et al., 2019). Intrinsic AMR genes, relatively stationary, in addition to producing a resistant phenotype, help regulate the physiology and metabolism of bacteria (Cox and Wright, 2013). Mobile AMR genes can rapidly spread by horizontal gene transfer occurring either through transformation, conjugation or transmission (Singh et al., 2019; von Wintersdorff et al., 2016). Mobile genetic elements—plasmids, integrons, transposons, genomic islands, are vehicles for transferring AMR genes in the gut microbiota (Table 2).

**Table 2:** Mechanism of antibiotic resistance.

S. No.	Mechanism	Description	Examples	References
1.	Enzymatic degradation	Bacteria produce enzymes that degrade the antibiotic	Beta-lactamases, carbapenemases	Bush (2018) and Bush and Jacoby (2010)
2.	Efflux pumps	Remove any potentially dangerous molecules from the anterior of the cell	RND and MATE	Ghotaslou et al. (2018) and Soto (2013)
3.	Target modification	Modification of antibiotic target	Methylation of 16S rRNA or 23S rRNA, MRSA (mecA gene)	Peterson and Kaur (2018)
4.	Reduced permeability	Changes in cell membrane permeability	Porin	Delcour (2009)
5.	Biofilm formation	Bacteria form biofilms that protect them from antibiotics and the immune system	*Campylobacter jejuni*	Buret and Allain (2023) and Grooters et al. (2024)
6.	Horizontal gene transfer	Transfer of ARGs between bacteria via plasmids, transposons, or phages	Conjugation, transformation, transduction	Groussin et al. (2021) and Huddleston (2014),
7.	Antibiotic modification	Enzymatic alteration of the antibiotic (phosphorylation, acetylation, and adenylation)	N-acetyl transferases, O-phosphotransferases, O-adenyltransferases	Peterson and Kaur (2018)

### 3.1 Mechanism

#### 3.1.1 Horizontal gene transfer

##### 3.1.1.1 Conjugation

Conjugation, known as bacterial sex, is a major horizontal gene transfer mechanism where the donor DNA is transferred to the recipient by direct contact via pilus or pore (Guglielmini et al., 2013; Virolle et al., 2020). Conjugation occurs through a series of events, including cell-to-cell contact, the formation of mating pairs, and the horizontal transfer of genetic material, such as plasmids or transposons, into the recipient cell’s cytoplasm (Peterson et al., 2011). Conjugative transposons integrate into new genome locations, facilitating genetic diversity and responsible for developing AMR and virulence (Salyers et al., 1995; Singh et al., 2019). Genetic flux through conjugation can be observed in inflammatory conditions like inflammatory bowel syndrome or infections caused by *E. coli* or *Salmonella* spp. (Stecher et al., 2012). The conjugation efficacy of the β-lactamase plasmid was reduced in research by Machado and Sommer (2014) when clinical isolates of *E. coli* were co-cultured with human intestinal cells that produce protein-based factors. They concluded that any damage to intestinal cells caused by toxins, drugs, or inflammation reduces the production of peptides, thereby promoting conjugation. A study revealed that a transitory intestinal colonization by an animal-derived *E. faecium* strain that carries mobile elements with the *vanA* gene resistance to a human-derived *E. faecium* isolate poses a risk of infection, particularly in immunocompromised patients (Lester et al., 2006).

Rooney et al. (2019) used a triple stage chemostat model of the human gut to demonstrate the colonization, clonal expansion, and transfer of CRE genes from *Klebsiella pneumoniae* to the microbiota of CRE-negative human faeces.

A mouse model with human-derived microbiota was created in order to evaluate the conjugative transfer of ARGs by *E. coli* utilizing fluorescently labeled protein in the gut without the use of antibiotic selection pressure. According to their findings, the ARG-bearing RP4 plasmid from *E. coli* spread to a variety of bacterial taxa, and the model can be used to comprehend the prerequisites for gene transfer and conjugation (Sher et al., 2025).

Factors such as biofilm formation, the density of donor or recipient bacteria, environmental conditions (availability of nutrients, pH, temperature), exposure to medications and preservatives decides the rate of conjugation (Liu et al., 2023). According to the study, the level of antibiotic-induced dysbiosis affects the colonization of *Salmonella* species in the gut and the conjugative transfer of the multi-drug resistant *IncA*/*C* plasmid to commensal *E. coli*. They also came to the conclusion that using antibiotics ethically is crucial because the latter may cause the dissemination of ARG (Yilmaz et al., 2024). The antibiotic resistance profile of mucin-degrader *Akkermansia muciniphila*, ubiquitously present in the adult human gut microbiota is poorly understood. Recent studies revealed resistance to quinolones and horizontal gene transfer of sulphonamide and aminoglycoside resistance genes from *Salmonella enterica*, indicating the need to access the spread of ARGs (Guo et al., 2017).

##### 3.1.1.2 Transformation

It refers to the ability of the bacterial cell to uptake and integrate extracellular DNA enabled by bacterial competence (Finkel and Kolter, 2001). The primary catalyst for transformation in gut microflora includes conditions like nutrition competition or DNA repair as a result of antibiotic damage (Finkel and Kolter, 2001; Huddleston, 2014). Extracellular DNA maintains the structural integrity of intestinal biofilms, suggesting transformation a crucial mechanism for bacterial persistence and adaptation in the gut environment (Licht et al., 1999).

According to Chowdhury et al. (2024), *Enterococcus fecium* developed kanamycin resistance by transformation in the presence of antibiotics, demonstrating that bacteria in the gut can absorb eARGs from their surroundings. According to the findings, the degree of gene uptake is correlated with antibiotic levels, suggesting that resistance gene acquisition may be facilitated by higher antibiotic concentrations.

##### 3.1.1.3 Transduction

The transfer of bacterial DNA through bacteriophages and are classified into generalized transduction and specialised transduction (Thierauf et al., 2009). There is little knowledge regarding the transmission of the AMR gene by bacteriophages in the gut. In transduction, phages can transfer genes between bacteria without requiring coexistence and can cross taxonomic boundaries (Muniesa et al., 2013). ARG-carrying phages are prevalent in the human gut and other environment and the number rises following an antibiotic exposure (Fernández-Orth et al., 2019). Studies conducted on mouse models have demonstrated that transduction drives genetic diversity in *E. coli* strains that colonize the gut and can lead to the development of drug resistance in gut bacteria (Frazão et al., 2019).

**Figure 1:**
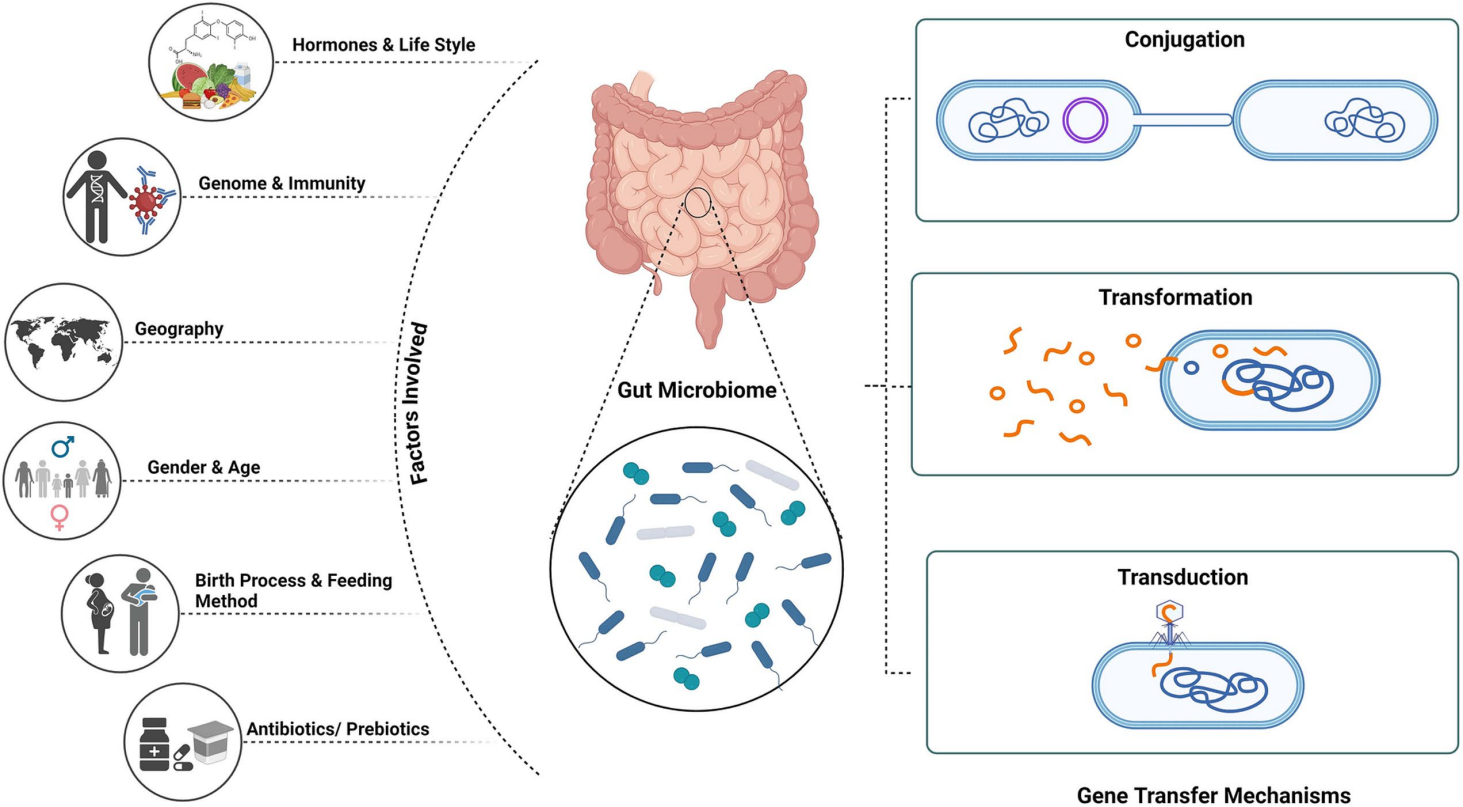
Mechanism of gene transfer and factors affecting gut microbiome. Created using BioRender.com.

Studies have reported that on treatment with β-lactam antibiotics, the expression of phage encoded genes in *Staphylococcus aureus*, responsible for encoding proteins that regulate cell wall metabolism, stress are upregulated (Maiques et al., 2006). Antibiotic treatment results in the abundance of phage-encoded AMR genes increasing the spread within the gut microflora (Modi et al., 2013). For instance, *Streptococcus pyogenes emm12* resistance has emerged in multiples due to the phage element *Φ HKU.vir*, which carries the superantigen gene *ssa* as well as the *spec* and *DNase* genes *spd1* (Davies et al., 2015). In metagenomic research, crAssphage—one of the most prevalent phages in the human gut—has been employed as a marker for faecal contamination. The abundance of resistance genes in the environment must be related to faecal contamination rather than environmental selection, according to Karkman’s et al. (2019) analysis. Therefore, in order to prevent erroneous assumptions regarding environmental selection for antibiotic resistance, the degree of faecal contamination must be taken into account (Dutilh et al., 2014; Karkman et al., 2019) (Figure 1).

#### 3.1.2 Antibiotic and target modification

Exposure protection, a common method of resistance, prevents antibiotic exposure in adjacent sensitive cells by allowing specific bacterial species to degrade antibiotics. The degradation of the antibiotic reduces antibiotic concentrations, which can benefit neighbouring susceptible cells is well recognized and demonstrated using various antimicrobial compounds (Gjonbalaj et al., 2020; Pathak et al., 2023).

The gut microbiota also regulates antibiotic absorption by metabolizing the drug or modifying the intestinal environment, resulting in variations in drug bioavailability, affecting their efficacy and toxicity. Certain bacteria in the gut, for example, can metabolize beta-lactam antibiotics such as penicillin by releasing β-lactamases, rendering them inactive and reducing their potency (Ramirez et al., 2020). The *cfxA*, *cfiA*, and *cepA* genes are associated with resistance to β-lactam antibiotics, while the *tetQ* gene is associated with resistance to tetracyclines (Lamberte and van Schaik, 2022).

Vancomycin’s interaction with the gut microbiota is one of the most important instances showing how the gut microbiota influences the choice of antibiotic therapy. Vancomycin’s pharmacokinetics and pharmacodynamics can be influenced by the gut microbiota through changes in its distribution, metabolism, and absorption, as well as its capacity to trigger an immunological response. Moreover, vancomycin-induced dysbiosis of the gut microbiota has been linked to heightened vulnerability to *Clostridium difficile* infection.

Harris et al. (2000) shown that animals express a variety of catecholamine-degrading enzymes throughout the GI tract, particularly in the colon, where the gut microbiome is most abundant.

A study has demonstrated that the intestinal microbiota’s diversity is significantly diminished for at least 28 days following a single dosage of clindamycin, with an ongoing loss of almost 90% of the usual microbial taxa from the cecum. Prior to antibiotic treatment, a fraction of bacterial taxa that contributed only slightly to the microbial consortium experienced rapid sequential expansion and contraction due to the loss of microbial complexity (Buffie et al., 2015).

Adenylyltransferases (ANT) catalyze the adenylation of a hydroxyl group in response to ATP, O-phosphotransferases (APH) catalyze the phosphorylation of a hydroxyl group in response to ATP, and N-acetyltransferases (AAC) catalyze the acetyl-CoA-dependent acetylation of an amino group. These three types of enzymes are known to modify aminoglycosides (Shete et al., 2017). An investigation found that enterococcal isolates had a high frequency of genes modifying aminoglycosides (Shete et al., 2017).

Additionally, bacteria can alter the molecular targets of antibiotics, causing minor structural changes that disrupt the highly precise interaction between the antibiotic and its target molecule. For instance, mutations in 23S rRNA confer resistance to macrolides, lincosamides, and streptogramin B; mutations in DNA topoisomerase II and IV result in resistance to quinolones and fluoroquinolones; and mutations in penicillin-binding proteins decrease the effectiveness of β-lactams. Through the efflux proteins found in their cell membrane, bacteria are able to pump out antimicrobial substances. The majority of these proteins are multidrug transporters, while some may be antibiotic-specific. Reduced permeability of the outer membrane, which lowers antibiotic absorption, is another mechanism of resistance (Ramirez et al., 2020).

#### 3.1.3 Efflux pumps

Efflux pumps actively transport antibiotics out of bacterial cells, lowering their intracellular concentrations and leading to multidrug resistance (Gaurav et al., 2023). The ATP-binding cassette (ABC) superfamily, the major facilitator superfamily (MFS), the multidrug and toxic compound extrusion (MATE) family, the resistance nodulation cell division (RND) family, the small multidrug resistance (SMR) family, and the proteobacterial antimicrobial compound efflux (PACE) family are the six main families of efflux pumps that have been identified in bacteria thus far (Gaurav et al., 2023).

The *E. coli* genome contains around 20 drug efflux system genes. Previously unknown, *E. coli* cells survive in the intestine, which has a low oxygen concentration. Anaerobic conditions dramatically increase the expression of the MdtEF drug efflux system in *E. coli*, and the resulting increase in drug efflux activity results in MDR (Nishino et al., 2021).

Biofilms, as opposed to their planktonic state, are organized group of microorganisms that reside in a matrix of extracellular polymeric substance (EPS) that they produce. They form colonies by adhering to one another on living or non-living surfaces, and they differ in their rates of growth and gene expression (Rather et al., 2021). Additionally, species that are essential for a healthy gut mucosa form biofilm, which can help the host by strengthening defenses, lengthening the time bacteria stay in the body, improving nutrient exchange between the microbiota and the host, increasing plasmid transfer rates, expressing colonization factors, and indicating resistance to colonization by a healthy mucosal biofilm (Miller et al., 2021; Tytgat et al., 2019).

*B. thetaiotaomicron* accounts for 12% of the gut microbiota and 6% of the faecal microbiome. *B. thetaiotaomicron* has been found to break down sugar moieties in food particles and in the mucus layer, indicating that biofilm production may play a significant role in their way of life. As a result, biofilms in the human gut can be useful or harmful to the host, depending on whether they are formed by commensal microbiota or enteric pathogens (Béchon and Ghigo, 2022).

Most clinically utilized antibacterial medicines must permeate one or both of the cell envelope membranes in order to reach their required site of action, such as the outer leaflet of the Outer membrane. Loss of porins and other transport systems might alter a drug’s overall capacity to pass through this membrane, which can result in clinical antibacterial resistance, especially in Enterobacteriaceae. Mutations in porin expression reduce expression, limiting nutrients and mediating resistance in bacteria (Masi et al., 2017).

### 3.2 The progression of colonization and microbial resistance

Overuse of antimicrobial medications, especially in immunocompromised individual, increases the risk of infection from opportunistic pathogens and result in the development of MDR bacteria in the gut microbiome (Dethlefsen and Relman, 2011). Common antimicrobial resistance genes, that are resistant to tetracycline, vancomycin, bacitracin, cephalosporin, and the macrolide-lincosamide-streptogramin (MLS) group have been found in the gut microbiomes globally (Forslund et al., 2014). Gut microbiota plays an important role in host defence by preventing exogenous bacteria and facilitating the growth of indigenous bacteria (Pilmis et al., 2020). This defensive role, referred as colonization resistance, is disturbed by the inappropriate use of broad-spectrum antibiotics (Nasiri et al., 2018; Pilmis et al., 2020). Studies have shown that, oral streptomycin administration altered the gut microbiota in mice increasing the susceptibility to *Salmonella* infections, with similar findings observed in other animal and human studies (Bartosch et al., 2004; Pecquet et al., 1991; Pilmis et al., 2020).

### 3.3 Mechanisms responsible for colonization resistance

**Figure 2:**
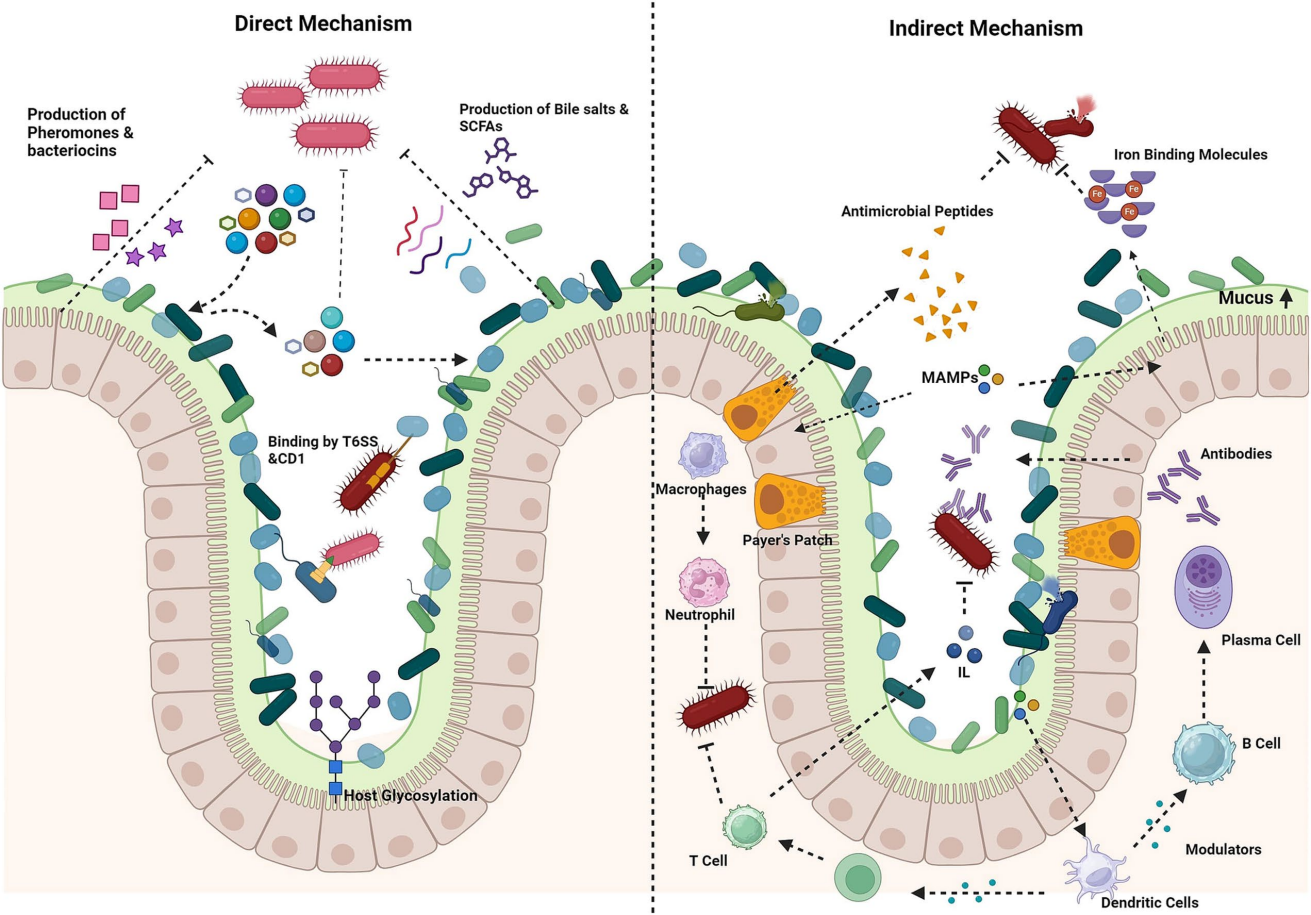
Mechanism of colonization resistance. Direct mechanisms: production of antimicrobial compounds (e.g., bacteriocins, short-chain fatty acids, bile salts), nutrient competition and host glycosylation of epithelial cells by bacteria, for nutrient adhesion, directly kill pathogens via contact-dependent inhibition (CDI), the type VI secretion system (T6SS) or secreted molecules. Indirect mechanisms: stimulation of mucin production by goblet cells forms a protective barrier. Microorganism-associated molecular patterns (MAMPs) trigger the secretion of antimicrobial peptides, which prime macrophages and dendritic cells. Macrophages inhibit pathogens through phagocytosis and the release of reactive oxygen species (ROS). Dendritic cells activate T cells, leading to the activation of immune responses and the stimulation of interleukin production. In Peyer’s patches, dendritic cells stimulate B cells to produce antibodies. Iron-binding proteins limit free iron availability to pathogens. Created using BioRender.com.

Colonization resistance, mediated by various mechanisms, is a process where the commensals in a healthy gut from the upper proximal to the intestine guard the host from pathogen invasion (Ducarmon et al., 2019; Ke et al., 2023; Kim et al., 2017). This mechanism was discovered when the depletion of the commensal bacteria due to antibiotic treatment increases the vulnerability to enteric pathogens. The gut microbiota aids in the process by synthesizing and secreting over 500,000 metabolites into the lumen (Chang, 2020). Although the mechanisms underlying colonization resistance are poorly understood, they can be broadly divided into direct and indirect mechanisms (Ducarmon et al., 2019; Khan et al., 2021) (Figure 2).

#### 3.3.1 Direct mechanism

The microbiota encourages direct colonization resistance, through antagonism and resource competition. Using variety of mechanisms, bacteria compete for both limited physical space and scarce nutrients. Closely related bacterial species that occupy same niches or resources tend to outcompete (Pickard et al., 2017).

##### 3.3.1.1 Nutrient competition

The nutrient niche theory, proposed by Rolf Freter in 1983, states that microorganisms will colonize, multiply and utilize the nutrients as per their requirements. The gut microbiota has a unique nutritional ability enabling it to digest resistant starch, cellulose, inulin, pectin, mucus and bile salts into carbon and nitrogen sources support their growth. Pathogens must compete with gut commensals for nutrition and to colonize (Horrocks et al., 2023; Pickard et al., 2017).

Commensals generally alter the pathogens virulence factor directly by the production of metabolites (Khan et al., 2021). Studies have shown that commensal *E. coli* with EHEC competes for amino acids, organic acids, and other nutrients (Fabich et al., 2008; Leatham et al., 2009). SCFA such as, butyrate, downregulates the expression of secretion system type 3 proteins (SST3) in *Salmonella enteritidis* and *Salmonella* Typhimurium (Gantois et al., 2006). Inhibiting the pathogen growth and colonization requires phylogenetically diverse species to prevent nutrient access and establish colonization resistance (Spragge et al., 2023).

Commensal species have evolved metabolic pathways to utilize mucins and dietary carbohydrates as key intestine nutrition source (Kim and Ho, 2010; Kamada et al., 2012). *Citrobacter rodentium* and *E. coli* may compete for monosaccharides while mutualistic *Bacteroides* species secrete sialic acid and fucose from host glycans, which are essential sugar source for *Salmonella* Typhimurium and *C. difficile* invasion. These carbohydrates are only accessible to pathogens when antibiotic therapy decreases the commensal population (Ng et al., 2013; Pickard et al., 2017). Bacterial development requires iron, a crucial trace metal that even the host firmly holds, especially during an inflammatory response. Through siderophores, *Salmonella* Typhimurium scavenge the host and commensal requirement for iron throughout an infection (Ducarmon et al., 2019; Sassone-Corsi and Raffatellu, 2015). An efficient way to lessen the severity of a Salmonella infection is to use immunization strategies against siderophores (Sorbara and Pamer, 2019). According to studies, two *Klebshiella* species—*K. oxytoca* and *K. michiganensis*—provide colonization resistance against *Enterobacteriaceae* that are resistant to antibiotics by means of nutritional competition. Colonization resistance was associated with resource utilization, and *Klebshiella species* reduced the colonization of *E. coli* and *Klebshiella pneumoniae* in mice and *ex vivo* investigations (Horrocks et al., 2023). When the commensal gut microbiota reduces dietary amino acids, it has been shown to increase resistance to *Citrobacter rodentium* colonization (Caballero-Flores et al., 2020).

##### 3.3.1.2 Bacteriocin

Bacteriocins are short, toxic ribosomal synthesized antimicrobial peptides produced by specific bacterial species that can inhibit the colonization and growth of other species. Their mechanisms of action are multiple including disturbing RNA and DNA metabolism, pore formation in the cell membrane, influence on protein and DNA synthesis (Benítez-Chao et al., 2021; Ducarmon et al., 2019; Pilmis et al., 2020). Peptides are categorized into post-transduction modified (type I) and unmodified peptides (type II), are typically effective against closely related bacteria and exhibit strong specific activity against clinical targets (including MDR strains) (Cotter et al., 2013). Many bacteriocins, from the lactic acid bacteria, human and animal gut microbes, and probiotics like *Bifidobacteria*, would engage in gastrointestinal competition (Hammami et al., 2013). It has been discovered that the Sactibiotic thuricin CD (bacteriocin type I) is effective against *C. difficile*. While sactibiotic, subtilosin A, exhibits efficacy against *Listeria monocytogenes*, *Streptococcus pyogenes*, and *Enterococcus faecalis*. In contrast, *Pediococcus acidilactici* MM33 secretes pediocin PA-1 (bactericin type II), that act against vancomycin-resistant *Enterococci* (VRE) colonization in the gut (Pilmis et al., 2020). The extent to which bacteriocins contribute to colonization resistance to pertinent intestinal pathogens is still unknown while they support ongoing intraspecies competition in the gut (Pickard et al., 2017). *Pediococcus acidilactici* produces bacteriocins that hinder the growth of planktonic cells of *Salmonella* Typhimurium in addition to preventing the formation of biofilms. *Probiotic Bacilli*, on the other hand, generate bacteriocins such subtilin and subtilosin A, which particularly prevent *Salmonella* from forming biofilms without harming the planktonic cells (Deng and Wang, 2024).

##### 3.3.1.3 Type VI secretion system

Type VI secretion system (T6SSs) is a mechanism by which bacteria transport proteins into or out of target cells during infection, facilitating interbacterial competition (Russell et al., 2011).

Enteric pathogens use T6SSs to antagonize symbiotic gut *E. coli*, facilitating colonization and disease progression. T6SS loci are also widely distributed in human gut *Bacteroidales* including *Bacteroides*, *Parabacteroides*, and *Prevotella*, and exist in three forms: GA1, GA2, and GA3 (Coyne and Comstock, 2019).

The GA1 and GA2 T6SS loci can be transferred between many intestinal species and Bacteroidales families, however the GA3 T6SSs are exclusive to *Bacteroides fragilis*. The GA3 T6SSs are the only ones that have been demonstrated to target almost every type of Bacteroidales found in the gut (Coyne and Comstock, 2019). Numerous studies have discovered the existence of a T6SS and its related effectors and immune proteins that significantly influences the competitiveness between as it involves a variety of effector and immune protein combinations, and can have a wider target range (Pickard et al., 2017).

#### 3.3.2 Indirect mechanism

Indirect colonization resistance is facilitated by the host-commensal flora interaction, by maintaining the epithelial barrier, regulation of bile acid metabolism, and production of antimicrobial peptides (RegIII and angiotensin-4) (Pilmis et al., 2020).

##### 3.3.2.1 Antimicrobial peptide production

Antimicrobial peptides (AMPs), recognized as a crucial line of defence against infections, are produced by all life forms (Pilmis et al., 2020). AMPs have a multiple mechanism of action, targeting peptidoglycan and bacterial cell membrane (Mookherjee and Hancock, 2007). Bacterial membranes, composed of cardiolipin and phosphatidylglycerol are negatively charged, interact with the positively charged antimicrobial peptides leading to lysis (Pilmis et al., 2020). The host (epithelial and paneth cells) requires taurine or lipopolysaccharide to produce ANG-4 (ribonuclease) and RegIII (type C lectin). Furthermore, the gut bacterium *Bacteroides thetaiotaomicron* induces ANG-4 expression, which has bactericidal effect against both Gram-negative and Gram-positive bacteria (Pilmis et al., 2020). Lipopolysaccharide-stimulated Toll like receptors (TLRs), notably TLR-4, in the microbiome can trigger RegIII production (Mukherjee et al., 2014). Flagellin also activates the TLR-5 and TLR7 receptors on dendritic cells resulting in the release of IL-23, which prompts innate lymphoid cells to release IL-22, increasing the synthesis of RegIII (Pilmis et al., 2020). Commensal bacteria activate MyD88 signaling in paneth cells and other epithelial cells, which in turn promotes the synthesis of the antimicrobial lectin regenerating islet-derived protein 3γ (REG3γ). By preventing *Salmonella* Typhimurium from penetrating host tissues, this antimicrobial response promotes gut health and prevents infection (Deng and Wang, 2024).

##### 3.3.2.2 Bile acid metabolism

Bile acids, produced by the liver to breakdown dietary lipids, have antibacterial characteristics. Primary bile acids are linked with glycine or taurine to improve solubility (Ducarmon et al., 2019). Bile acids exhibits dual role in microbial growth where primary bile salts influence germination and vegetative growth of *C. difficile* spores and Secondary bile acids have been discovered to prevent the growth (Sorg and Sonenshein, 2008). For instance, the symbiotic microbe *Clostridium scindens* can change the main bile acids (cholic acid and chenodeoxycholic acid) into the secondary bile acids (deoxycholic acid and lithocholic acid). Thus, in both animals and humans, *C. scindens* increases resistance to *C. difficile* infections in a secondary bile acid-dependent manner (Buffie et al., 2015).

##### 3.3.2.3 Epithelial barrier maintenance

The inner and outer mucous layers, the epithelial barrier, and its associated immunological barrier make up the physical gut barrier. The inner mucus layer is impermeable and strongly adhered to epithelium thus restricting the movement of bacteria, preventing direct contact between host and commensal bacteria of gut microbiome, thereby avoiding inflammatory reaction (Pilmis et al., 2020). As the thickness of the mucus layer decreases it becomes more vulnerable to pathogen colonization. Therefore, a western-style diet poor in carbohydrates, antibiotic therapy, or other medications that have an impact on the microbiota, alters the thickness of the mucus layer increasing vulnerability to infection (Desai et al., 2016; Pilmis et al., 2020). The NF-κBpathway is activated by the gut bacteria when the mucus layer is altered, encouraging tissue healing by activating innate immunity receptors such as synthesis of anti-apoptotic proteins, increasing cell proliferation, stabilising tight junctions, negatively regulating the production of pro-inflammatory cytokines (Pilmis et al., 2020; Rakoff-Nahoum et al., 2004).

## 4 Screening for AMR

**Figure 3:**
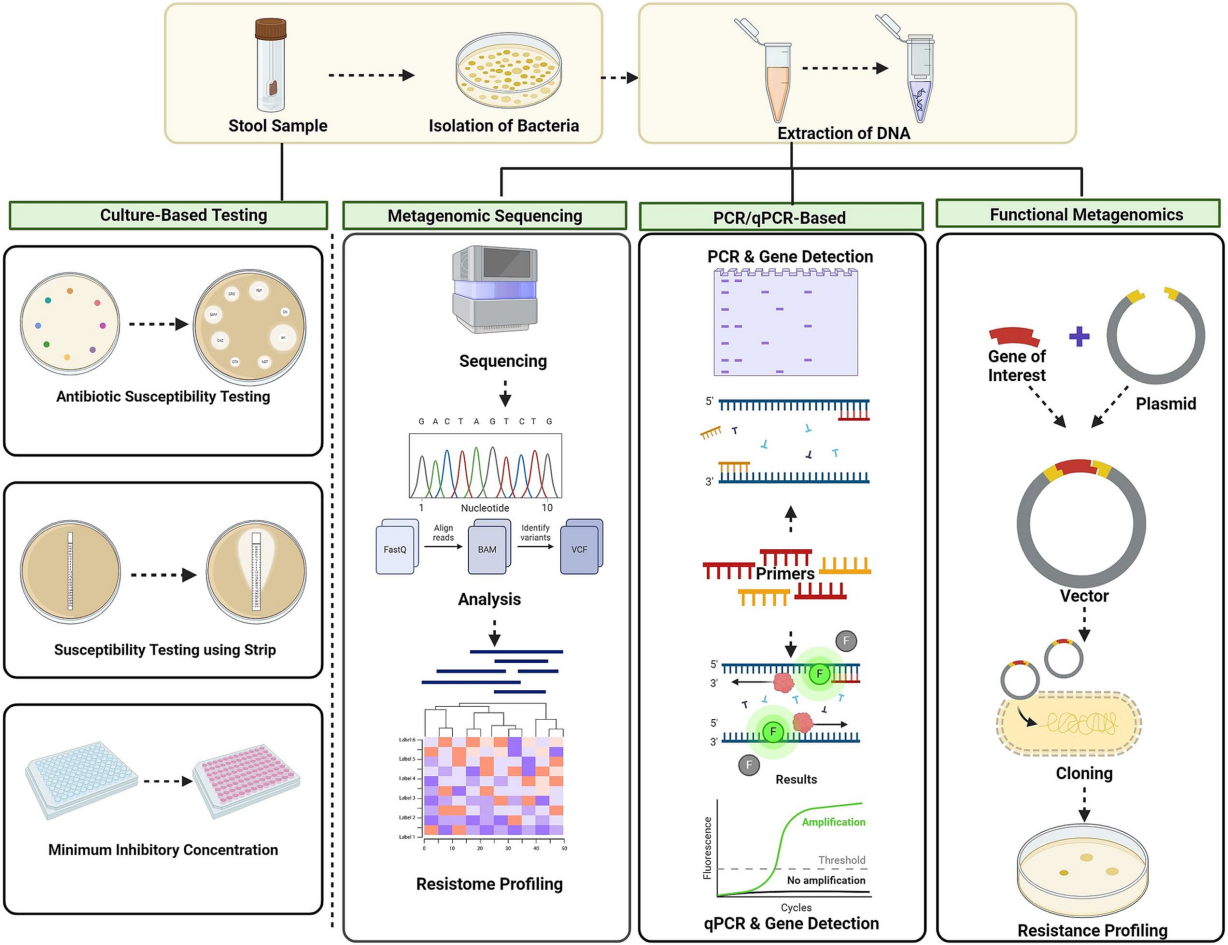
Techniques for AMR screening culture-based methods, metagenomic approaches, PCR-based techniques, and functional metagenomic approach. Culture-based methods involve disc diffusion, MIC. Metagenomic approaches: next-generation sequencing (NGS) and shotgun metagenomic sequencing. PCR-based techniques: conventional PCR and quantitative PCR (qPCR). Functional metagenomics: using vectors involves cloning resistance genes and screening for their functional traits. Created using BioRender.com.

Antibiotic resistance genes in the gut microbiota can be passed to other bacteria, increasing the risk of evolution of pathogenic strains (Hu et al., 2013; Theophilus and Taft, 2023). AMR, characterised using a variety of techniques, are necessary for the understanding and monitoring of a variety of resistance genes that can contribute to treatment failures and the spread of resistant infections, in complete environmental communities. The screening of AMR, concern for human health and socioeconomic development, helps in the better understanding of the ARGs and identification of novel ARGs (Theophilus and Taft, 2023) (Figure 3 and Table 3).

**Table 3:** Current and emerging techniques for AMR screening.

S. No.	Method	Description	Advantages	Limitations	References
1.	Metagenomic Sequencing	Sequencing of DNA to find ARGs	Comprehensive	Expensive, requires experts in bioinformatics	Bogri et al. (2024) and Merrick et al. (2023)
2.	qPCR	Quantifies specific genes using primers	Ability to test for multiple antibiotic genes, rapid	Lower specificity compared to culture and staining, expensive, limited to known targets	Liu et al. (2019)
3.	Culture-based methods	Isolates organisms in selective media and antibiotics	Provide reproducible results with minimal error, isolation of specific target organisms, screening at a range of antibiotic concentrations	Turnaround time, contamination	Figdor and Gulabivala (2008) and McLain et al. (2016)
4.	Functional metagenomics	Clones DNA into host to find ARGs	Identifies ARGs based on function, highly sensitive	Limited insert size, require experts	De (2019), Mullany (2014), and Yadav and Kapley (2021)
5.	Resistome profiling	Comprehensive analysis of all resistance genes in a sample	Predicting the possible resistance pattern, determine resistome	Requires advanced sequencing and bioinformatics, limited differentiation	Waskito et al. (2022)
6.	Nanopore sequencing	Real-time sequencing of long DNA fragments	Analysing sequences in real time, size, cost, simple library prep, and portability	High error rate, expensive	Delahaye and Nicolas (2021) and Jain et al. (2016)
7.	CRISPR-based detection	Uses CRISPR-Cas systems to detect specific DNA sequences	Simplicity, sensitivity, and specificity, robust result	Off-target effects, expert required, cost and accessibility	Kaminski et al. (2021), Mayorga-Ramos et al. (2023), and Shin et al. (2024)
8.	Single-cell genomics	Analyzes genetic material from individual cells	High resolution, identifies rare cells	Nosier and more variable data	Chen et al. (2019)
9.	Metaproteomics	Studies the protein composition of microbiomes	Direct functional insights, comprehensive analysis	Limited database, complex data analysis	Kleiner (2019) and Petriz and Franco (2017)

### 4.1 Culture based techniques

Culture-based analysis, as recommended by the European Committee on Antimicrobial Susceptibility Testing (EUCAST) and the Clinical Laboratory Standards Institute (CLSI), is the gold standard technique for detecting AMR in gut microbiota (Hassall et al., 2024). The diffusion assay and e-test minimum inhibitory concentration approach, part of the standard methods, detects bacterial growth in various antibiotic concentrations after being isolated from selective media (Yamin et al., 2023). Culture-based approaches have several benefits such as targeted isolation, reproducibility, cost effectiveness, quantitative and qualitative measurements (McLain et al., 2016). However, they can be potentially variability in the results, time consuming, limited options for antibiotic testing and ability to detect all potential resistance genes (Hassall et al., 2024). Due to these limitations, the evaluation of the antimicrobial susceptibility (AMS) patterns of the entire microbiome is restricted to indicator bacteria like *E. coli*, a reservoir of ARGs (Firoozeh et al., 2011; Firoozeh et al., 2013; Nyirabahizi et al., 2020; Penders et al., 2013; Neamati et al., 2015). Studies using *enterococci* or *E. coli* as markers have shed light on the occurrence of AMS throughout geographical regions, the effects of hospitalisation and population density, and the link between AMS in humans and food animals (Nyirabahizi et al., 2020). Culture-based analysis of AMS have shown the link between the AMS of faecal *E. coli* and that of *E. coli* implicated in diseases including urinary tract infections. A broad range of antibiotics like ampicillin (Amp), amoxicillin (Amx), aztreonam (Azt), cefotaxime (Cefo), ceftriaxone (Ceft), imipenem (Imp), meropenem (Mer), cefepime (Cef), piperacillin (Pip), vancomycin (Van), clindamycin (Cli), colistin (Col), polymyxin B (PolB), daptomycin (Dap), Fosfomycin (Fos), tetracycline (Tet), doxycycline (Dox) is used to test the commensals antimicrobial drug susceptibility. The minimum inhibitory concentration (MIC) of each antibiotic is determined by the observing the bacterial growth with the strip of chosen antibiotic (Bag et al., 2019). Culture-based studies have advanced the understanding of the relationship between antibiotic use and AMS of gut bacteria, revealing an association between antibiotic use and the prevalence of resistant faecal Gram-negative bacteria (Bruinsma et al., 2003; Murray et al., 1982; van der Veen et al., 2009). Individuals who have received medical treatment with antibiotics have been found to harbour these antimicrobial-resistant bacteria (Bartoloni et al., 2004; Grenet et al., 2004).

Other studies have found that people who do not have access to antimicrobial agents, such as those who live in remote areas, can develop faecal antimicrobial-resistant bacteria as a result of environmental exposure to organisms producing antibiotics (mould contaminated food), heavy metal contamination in drinking water (Bartoloni et al., 2004; Calomiris et al., 1984; Timoney et al., 1978).

### 4.2 Molecular diagnostics used for detection antimicrobial resistance

Molecular diagnostics have recently received attention due to their speed, accuracy, and independence from culture (Anjum et al., 2017). Advanced techniques like polymerase chain reaction assays, sequencing, and various genotyping approaches provide insights into the mechanisms of resistance transfer by identifying resistant-carrying integrons after antibiotic treatment (Geser et al., 2012; Gijón et al., 2012; Overdevest, 2011; van der Veen et al., 2009; Vinue et al., 2008; Vo et al., 2010). Following an outbreak, when phenotypic data is insufficiently precise to prevent potential outbreaks involving resistant bacteria, genetic characterization is sometimes employed as an indirect way to support epidemiological investigations. Also, local, national, or even international surveillance of AMR utilizes molecular characterization of AMR determinants (Anjum et al., 2017).

### 4.3 Metagenomics to characterize the AMR

Metagenomics involves the study of metagenomes or genetic material directly collected from environmental sources enabling the genomic analysis of every bacterium in a microbial ecosystem without individual identification (Lepage et al., 2013). Targeted PCR-based metagenomics, functional metagenomics, and sequence-based metagenomics are three different metagenomic methods that have been used to explore the resistome (Penders et al., 2013).

#### 4.3.1 PCR-based metagenomics

PCR and qPCR are widely used *in vitro* techniques that allow the exponential amplification of specific DNA and RNA sequences with high specificity, providing rapid means of identifying bacteria from various environments, including the detection of resistance genes (Galhano et al., 2021). Research have shown that transfer of resistant genes occurs within an ecosystem and across species, emphasizing the real-time applications of PCR-based metagenomics. The relative abundance of the resistant genes can be estimated by analysing the semi-quantitative result of qPCR (Knapp et al., 2011; Koike et al., 2007). PCR was utilized to detect the blaCTX-M gene variations in *E. coli* isolated from the human and chicken faecal sample, indicating the presence of ARGs in the gut microbiome (Valenzuela et al., 2023). The presence of *bla_CMY_* and *bla_SHV_* resistance genes in *E. coli* from migratory birds, indicated the potential for analysing gut microbiota resistance genes (Islam et al., 2022). Vien et al. (2012) using qPCR, showed that higher levels of Plasmid-mediated quinolone resistance genes in gut flora lead to fluoroquinolone resistance. Targeted PCR-based metagenomics remains a valuable technique for identifying the resistome due to the accessibility, and provides high-throughput analysis at reasonable costs. However, the fundamental drawback is that the data are skewed toward known resistance genes and pathways predominantly in case of convergent evolution, where a number of genes perform similar roles. Furthermore, a resistance gene with sequence variation found in numerous species may skew the results in favour of the species (Penders et al., 2013).

#### 4.3.2 Functional metagenomics

Functional metagenomics involves cloning DNA segments into a vector (like a plasmid) and expressing these segments in heterologous hosts, often *E. coli*. Transformants are cultivated on antibiotic-containing media to assess the expression of resistance genes, with findings relying on each gene’s ability to express in surrogate hosts, allowing for subsequent sequencing (Schmieder and Edwards, 2012). Functional metagenomics DNA screening was used to identify the reservoir for resistance in samples of faeces and saliva from two healthy people. Sequencing and annotation of clones exhibited resistance to 13 different antibiotics resulted in identification of 95 unique inserts encoding functional antibiotic resistance genes. Out of these, 10 previously unknown beta-lactamase gene families where identified indicating an underappreciated barrier separating these unique resistance-producing bacteria from common human pathogens (Schmieder and Edwards, 2012; Sommer et al., 2009). Additionally, a functional screening for seven antibiotics utilizing gut microbiome metagenomic libraries from healthy people revealed novel AMR genes against kanamycin, D-cycloserine, and amoxicillin (Cheng et al., 2012). A recent study using functional metagenomics identified three novel genes, TMSRP1, ABCTPP, and TLSRP1, responsible for the osmotolerance in human gut microbiota (Verma et al., 2018). Despite being commonly used, the functional metagenomics-based approach has several drawbacks. The method depends on individual gene’s ability to express itself in surrogate hosts, leading to false negative results if resistance genes that are not produced by the surrogate host because they require several regulatory elements, or posttranslational modifications. Also, the foreign gene may engage in unique interactions with the surrogate host’s cellular machinery, leading to false positives (Penders et al., 2013; Schmieder and Edwards, 2012).

#### 4.3.3 Sequence based metagenomics

Sequence-based metagenomics eliminates the requirement for culturing by directly sequencing DNA from an environmental sample once it has been extracted, fragmented, and size-separated. Resistance genes are recognized by comparing metagenomic sequences to global sequence databases. The transition from Sanger sequencing to next-generation sequencing technologies, including the Roche 454 sequencer, Illumina’s Genome Analyzer, and Applied Biosystems’ SOLiD system, has significantly reduced the cost of metagenomic sequencing initiatives by producing shorter contiguous reads, higher genome coverage, and fewer consumable costs (Niedringhaus et al., 2011). Sequence-based metagenomics is increasingly used to study the human gut microbiome, but not directly targeting the AMR genes. However, the *in-silico* identification of resistance components has been made possible by the uploading of these metagenomic libraries to public databases (Penders et al., 2013). The ratio of chromosomal and extra-chromosomal genomes will always be heavily in support of chromosomes, producing a tonne of redundant data when one is only interested in the extra-chromosomal metagenome (Li et al., 2012). Sequence-based metagenomics is often only useful for identifying known genes because it is difficult to discover sequences with little resemblance to known reference sequences (Penders et al., 2013; Schmieder and Edwards, 2012). Additionally, the expression of the discovered genes is not provided by sequence-based metagenomics. Contrarily, sequence-based metagenomics offers a large amount of data not just on AMR genes but also on the whole gene content, making it possible to determine the metabolic profile and community composition. These metagenomic sets of data in particular make it possible to examine which bacteria in the community possess specific functional genes (Penders et al., 2013). A metagenomic study using a sequence data of 2,037 samples concludes that the human gut resistome is influenced by geographical locations and to a lesser extent on the disease conditions (Qiu et al., 2020) According to the metagenome data from mice with UTIs, oral antibiotic therapy led to an enrichment of particular taxa and ARGs and a decrease in the overall diversity of gut microbes. The results of this model demonstrated that after 24 to 72 h of cipro and fosfo treatment, cross-resistance to several types of antibiotics emerged (Xu et al., 2020).

## 5 Impact of AMR on health

Antimicrobial resistance, a naturally occurring process, has been accelerated due to the inappropriate overuse of antibiotics and poor infection control practices (Salam et al., 2023). Greater patient mobility and movement of carriers have increased the risk of spread of resistant pathogens globally (Findlater and Bogoch, 2018). Delays in appropriate treatments prolong the infection, this in turn puts at risk the immediate contacts of those infected, including health professionals, but it also enhances the dissemination of resistance within communities. Longer duration of disease and treatment due to AMR leads to increased financial costs for families and healthcare systems (O’Neill, 2016; Dadgostar, 2019). Development of new antibiotics has reached an almost complete standstill; no new classes have been discovered after 1987 (Silver, 2011). Drugs for chronic illnesses like those for diabetes and hypertension may provide more profitable economic opportunities for pharmaceutical corporations than newer antibiotics due to their extensive usage in each patient and the lack of problems with resistance. Additionally, smaller pharmaceutical companies struggle to meet the strict requirements for clinical trials involving antibiotics. This puts the development of several potential new agents in danger (Jindal et al., 2015).

This misuse of antibiotics, both in public and private health care facilities, is very common in developing countries like India, where studies indicate that 45–80% of patients suffering from viral respiratory infections and diarrhoea were inappropriately provided with antibiotics without proper diagnosis (Jindal et al., 2015; Kumar et al., 2008). Moreover, the use of antibiotics in agriculture to improve the yield has increased the diversity and abundance of AMR genes in urban, agricultural, and environmental settings (Baquero et al., 2008; Nesme et al., 2014; Wright, 2010; Zhu et al., 2013).

Vaccination plays an important role in blocking the spread of infectious diseases. But as the vaccination rates decline, the unvaccinated population, such as children, and immunocompromised patients are susceptible to the infection, in-turn enhancing the reservoirs of pathogens, some of which may acquire resistance to antibiotics. To effectively address AMR, multiple strategies are necessary, which include enhancing antibiotic stewardship, investing in new drug development, and maintaining a high level of vaccination to prevent the spread of infectious diseases (Muhsen et al., 2012; Zhu et al., 2013).

## 6 Effects of AMR on environment

Environmental factors have a global impact on development of AMR. Drug-resistant microorganisms and resistance genes could spread into the environment through excreta, water bodies (Konopka et al., 2022; Singer et al., 2016). In agriculture, out of the total antibiotics given to animals, 30–90% are excreted through urine and faeces, leading to environmental pollution and the development of resistance (Berendsen et al., 2015). Animal manure has been identified as one of the significant vectors of both antibiotic-resistant bacteria and residual antibiotics that may persist in the environment (Sarmah et al., 2006; Udikovic-Kolic et al., 2014). Heavy metals also contribute to the dissemination of AMR, often present in WWTPs from urban sources like domestic and commercial effluents, vehicle emissions, and industrial activities. The contamination is further increased by the widespread use of disinfectants, textiles, and common household items containing metal nanoparticles, including those of titanium, copper, and silver. In addition, other metals, including Pb, Cu, Zn, and Cd, were utilized in agriculture and aquaculture as fertilizers and for insecticides, fungicides, and animal growth promotion, thereby producing an optimal ecological environment for the development of AMR.

## 7 Effect of AMR on economy

The economic burden of AMR includes both direct or indirect costs. These direct medical costs of AMR relate to treatments, including prescription drugs for the disease and hospitalisation costs. Indirect costs are essentially the wider consequences of increased sickness and mortality, leading to decreased productivity and reduced economic output (National Academies of Sciences, Engineering, and Medicine et al., 2018). According to the CDC reports, antibiotic resistance in the United States alone might result in a $1,400 rise in hospital costs for treating patients with any type of bacterial infection (Centers for Disease Control and Prevention, 2013; Thorpe et al., 2018). However, this can sharply rise to more than $2 billion per year. According to a number of estimates, AMR costs would range from $300 billion to over $1 trillion annually globally by 2050. Healthcare is directly impacted financially by AMR, as seen by increased resource use and high costs for complex and expensive treatments (Dadgostar, 2019).

## 8 Future perspectives

To combat AMR, new solutions are urgently needed. Faecal microbiota transplantation (FMT) is the most advance treatment to tackle AMR and other tactics (such probiotics and bacteriophages) as prospective substitutes for infection prevention solutions (Gargiullo et al., 2019). FMT involves the endoscopic or oral administration of tablet preparations to a patient’s colon for transferring the microbiota from a donor. FMT is being researched for additional uses, however it is now recognized as a clinically extremely successful therapy for persistent *Clostridioides difficile* infection. FMT has recently been taken into consideration for the elimination of antibiotic-resistant bacteria from their reservoir in the intestine (Pilmis et al., 2020). FMT has been recognized as an effective treatment for additional conditions linked to altered gut microbiome, including intestinal inflammatory diseases like IBD, in addition to its use for MDR infections (Paramsothy et al., 2017; Wang et al., 2016).

Prebiotics, non-digestible food ingredients, favourably influence one or more species of bacteria in the colon by increasing their growth and/or activity. In contrast, probiotics are isolated, live organisms that are given to the host in order to boost their health.

These products have the potential to restore the balance of the gut microbiota by encouraging the recolonization of species, either directly via the action of prebiotics or indirectly through the careful selection of bacterial species in probiotics (Pilmis et al., 2020). When healthy individuals are exposed to antibiotic therapy, human milk oligosaccharides, a typical example of a prebiotic, are known to assist in re-establishing the balance between Firmicutes and Bacteroidetes (Elison et al., 2016). According to Cochrane, use of probiotics have successfully used in prevention diarrhoea (Wei et al., 2018). Using *Lactobacillus rhamnosus*, patients with vancomycin-resistant *enterococci* were successfully decolonized in two randomised investigations, while the combination of *Lactobacillus bulgaris* and *Lactobacillus rhamnosus* had no effect on the colonization rate in the Gram-negative range (Salomão et al., 2016).

## 9 Conclusion

Antimicrobial resistance is spreading across the globe and is contributing to an increase in hospital-acquired infections, mortality, and expenditures. Presently, bacterial enteric infections continue to contribute significantly to the global illness burden. Very little is known about the failure of the gut microbiota to give colonization resistance against these enteropathogens, even though the virulence factors involved in infection for many infectious agents are well understood. When established in a human, drug-resistant bacteria and resistance genes could spread into the environment through human waste. The antimicrobial susceptibility among these strains in various hosts, through time, and in various geographic regions has been the focus of extensive prior study on marker gut bacteria. Culture-based investigations are still relevant in the modern era of molecular methods since they are required to determine antibiotic susceptibility. To learn more about the possibility of the human gut microbiome as an AMR reservoir, however, targeted, functional, or sequence-based metagenomics are needed. Strategies based on the microbiota should be considered for MDRO prevention and therapy. Faecal microbial transplantation is a very promising approach, particularly when tested treatment have failed. Faecal microbial transplantation has so far been proven to be reliable and effective. However, in order to use faecal microbial transplantation in MDR clinical therapy, RCTs are required to standardise the methodology and establish regulatory parameters.
